# Detection of Microstructural Medial Prefrontal Cortex Changes Using Magnetic Resonance Imaging Texture Analysis in a Post-Traumatic Stress Disorder Rat Model

**DOI:** 10.3389/fpsyt.2022.805851

**Published:** 2022-04-21

**Authors:** Shilei Zheng, Han Wang, Fang Han, Jianyi Chu, Fan Zhang, Xianglin Zhang, Yuxiu Shi, Lili Zhang

**Affiliations:** ^1^Department of Radiology, The First Affiliated Hospital of Jinzhou Medical University, Jinzhou, China; ^2^Medical Imaging Center, Taian Central Hospital, Taian, China; ^3^Post-Traumatic Stress Disorder Laboratory, Department of Histology and Embryology, Basic Medical Sciences College, China Medical University, Shenyang, China; ^4^Department of Neurology, The First Affiliated Hospital of Jinzhou Medical University, Jinzhou, China; ^5^Department of Stomatology, The First Affiliated Hospital of Jinzhou Medical University, Jinzhou, China

**Keywords:** post-traumatic stress disorder, magnetic resonance imaging, radiomics, texture analysis, medial prefrontal cortex, single prolonged stress

## Abstract

**Background:**

Radiomics is characterized by high-throughput extraction of texture features from medical images and the mining of information that can potentially be used to define neuroimaging markers in many neurological or psychiatric diseases. However, there have been few studies concerning MRI radiomics in post-traumatic stress disorder (PTSD). The study's aims were to appraise changes in microstructure of the medial prefrontal cortex (mPFC) in a PTSD animal model, specifically single-prolonged stress (SPS) rats, by using MRI texture analysis. The feasibility of using a radiomics approach to classify PTSD rats was examined.

**Methods:**

Morris water maze and elevated plus maze were used to assess behavioral changes in the rats. Two hundred and sixty two texture features were extracted from each region of interest in T2-weighted images. Stepwise discriminant analysis (SDA) and LASSO regression were used to perform feature selection and radiomics signature building to identify mPFC radiomics signatures consisting of optimal features, respectively. Receiver operating characteristic curve plots were used to evaluate the classification performance. Immunofluorescence techniques were used to examine the expression of glial fibrillary acidic protein (GFAP) and neuronal nuclei (NeuN) in the mPFC. Nuclear pycnosis was detected using 4′,6-diamidino-2-phenylindole (DAPI) staining.

**Results:**

Behavioral results indicated decreased learning and spatial memory performance and increased anxiety-like behavior after SPS stimulation. SDA analysis showed that the general non-cross-validated and cross-validated discrimination accuracies were 86.5% and 80.4%. After LASSO dimensionality reduction, 10 classification models were established. For classifying PTSD rats between the control and each SPS group, these models achieved AUCs of 0.944, 0.950, 0.959, and 0.936. Among four SPS groups, the AUCs were 0.927, 0.943, 0.967, 0.916, 0.932, and 0.893, respectively. The number of GFAP-positive cells and intensity of GFAP-IR within the mPFC increased 1 day after SPS treatment, and then decreased. The intensity of NeuN-IR and number of NeuN-positive cells significantly decreased from 1 to 14 days after SPS stimulation. The brightness levels of DAPI-stained nuclei increased in SPS groups.

**Conclusion:**

Non-invasive MRI radiomics features present an efficient and sensitive way to detect microstructural changes in the mPFC after SPS stimulation, and they could potentially serve as a novel neuroimaging marker in PTSD diagnosis.

## Introduction

Post-traumatic stress disorder (PTSD) is a trauma and stressor-related disorder that results in complex somatic, cognitive, affective, and behavioral effects after exposure to traumatic events (1, 2). The typical symptoms of PTSD include re-experiencing trauma, increased alertness, persistent avoidance, and negative alterations in cognition or emotion (2, 3). While for the majority of individuals, these symptoms resolve within several weeks or months after exposure to trauma, ~10–20% of individuals experience PTSD symptoms that persist and are associated with impairment (4). In recent years, owing to the frequent occurrence of severe stress events, such as military combat, serious natural disasters, notable diseases, and major traffic accidents, the incidence of PTSD has increased (5).

The coronavirus disease 2019 (COVID-19) pandemic has posed unprecedented challenges to healthcare systems worldwide, and a relatively high proportion (7–53.8%) of PTSD has been reported in the general population during the COVID-19 pandemic in countries including China, Turkey, Spain, Italy, Nepal, Iran, the United States, and Denmark (6, 7). PTSD is closely associated with a patient's physical or psychological health problems and social dysfunction (8, 9), and it has attracted increasing attention from many research fields.

At present, the diagnostic criteria and assessments for PTSD rely on clinical interviews or subjective reports from the affected subjects, and occasionally on relevant collateral information from patients' intimates, using the fifth edition of the Diagnostic and Statistical Manual of Mental Disorders (DSM-5) or comparable criteria (10). Despite study having suggested that the DSM-5 description is an improvement over the previous model, it still may not represent the true underlying factor structure of PTSD (11). Due to the heterogeneity of patients, their differing experiences of different traumatic events, and different diagnostic criteria, the findings of pertinent studies are difficult to generalize and interpret with clinical significance (12). In addition, a reliable assessment is closely correlated with the experience of the assessing clinician (1).

In neuroimaging, magnetic resonance spectroscopy (MRS), resting-state functional magnetic resonance imaging, voxel-based morphometry, and diffusion tensor imaging have demonstrated abnormalities and dysfunction in different brain regions in PTSD patients (13–17). A previous MRS study conducted by us found that neurometabolite abnormalities in the amygdala and hippocampus were involved in a PTSD rat model (18). It is unfortunate that the limitations of these MRI techniques, such as hardware or software limitations, accuracy, reproducibility, or complicated sequences, have seriously hindered their clinical application in the field of PTSD. As a useful and valuable radiological examination, conventional MRI [T1- or T2-weighted (T2W) imaging] is widely used in clinical neurology. However, conventional MRI has little value for the diagnosis of psychiatric diseases such as PTSD, and there are currently no objective diagnostic tests available.

The term radiomics, which was first introduced by Lambin et al. (19), refers to the high-throughput processing and analysis of quantitative data extracted from medical images with a view to discovering meaningful associations between these data and particular disease features. Radiomics has been widely applied in the management of a wide range of cancers, and it can improve the accuracy of diagnosis, evaluate therapeutic effects, and assess overall prognosis (20–25). Furthermore, emerging clinical and experimental studies have shown that MR texture analysis (MRTA) can be used to as neuroimaging markers for many neurological diseases (26–31); it can provide an early, non-invasive, and accurate method to detect many neurodegenerative diseases because it can detect unseen or subtle signal changes and thus obtain latent image information (32–35). Sørensen et al. (36) has indicated that the major hallmarks (neurofibrillary tangles and amyloid-bpeptide) cannot be observed with conventional MRI in the early stages of Alzheimer's disease (AD), but their accumulated effects on the brain tissue can cause changes in image pixel distribution or intensity. These changes can form certain texture patterns in MRI images, and these can be captured by texture analysis (36). Our previous study showed that the differences in texture features in the neostriatum between Parkinson's disease (PD) patients and healthy controls and radiomics signatures based on conventional T2W images possessed good diagnostic performance for PD (37). However, there have been few studies concerning MRTA in PTSD, and it is not yet clearly understood whether a medial prefrontal cortex (mPFC) radiomics approach based on T2W images can be used to classify PTSD in rats.

The mPFC, which is receives extensive projections from limbic regions and seems to be a brain region that is highly sensitive to stress- or anxiety-related behaviors (38). Neuropathological studies have shown that alterations of structure and function in the mPFC can lead to decreases in the ability of the mPFC to regulate the amygdala, leading to over-response to fear stimulation in the amygdale (17, 39). Moreover, the ventral hippocampus-mPFC afferent pathways will further affect the encoding and updating of spatial cues during spatial working memory (40). Many MRI studies have revealed evidence of decreased mPFC volume and gray-matter signal in PTSD patients, and a significant correlation has been found between mPFC volume and the severity of PTSD symptomatology (39, 41–43). The mPFC plays a crucial role during the occurrence and development of PTSD; furthermore, structural changes in and dysfunction of the mPFC are important factors leading to memory disorder and fear memory in PTSD patients.

In the past decade, we have been committed to studying the pathogenic mechanisms of PTSD, and we have achieved some interesting results. In previous studies, we discovered that pathological changes of in neural cells, such as apoptosis and autophagy, may induce mPFC atrophy and dysfunction in single prolonged stress (SPS)-exposed rats (44–47). Acknowledging these findings, we speculate that cellular abnormalities may be associated with changes in MRTA results of the mPFC, and furthermore that MRTA could be used as a potential neuroimaging marker in clinical diagnosis of PTSD. The specific aims of present study were to compare the microstructural results obtained by MRTA to the cellular results obtained by immunofluorescence analysis, appraise changes of the microstructure of the mPFC of PTSD rats by using MRTA, and investigate the feasibility of using a radiomics approach based on conventional T2W images to classify PTSD rats.

## Materials and Methods

### Animals and Grouping

A total of 66 male Wistar rats (220–250 g) were housed with free access to food and water in an environment maintained at 22 ± 1°C on a 12/12-h light/dark cycle. Rats were randomly divided into five groups according to the time since SPS exposure: control (*n* = 18), 1 day post-SPS (SPS 1d; *n* = 10), 4 days post-SPS (SPS 4d; *n* = 10), 7 days post-SPS (SPS 7d; *n* = 18), and 14 days post-SPS (SPS 14d; *n* = 10). Eight rats each from the control and SPS 7d groups were used for behavioral tests, and ten rats per group were used for MRI examination and immunofluorescence. Rats in the control group remained in their home cages with no handling, and the SPS rats underwent the SPS procedure on the first day.

### SPS Procedure

In the present study, the PTSD rat model was established using the SPS procedure developed by Liberzon et al. (48). Briefly, rats were restrained for 2 h in a plastic container and then forced to swim in a plastic tub for 20 min. After a 15 min rest, the rats were then exposed to ether anesthesia until consciousness was lost (as tested by the loss of toe- and tail-pinch responses). After induction of general anesthesia, rats were placed in their home cages and fed routinely.

### Morris Water Maze (MWM) Test

In the MWM tests, each rat was placed in water facing the wall of the pool, with the drop location changing for each trial. They were then allowed 120 s to locate the submerged platform, where each was allowed to remain for 20 s. If a rat failed to find the platform within 120 s, it was guided gently onto the platform and allowed to stay there for 20 s. The tests lasted 5 consecutive days with four trials on each day. The escape latency time (ELT) was noted as an index of their learning capabilities. In the spatial probe tests, each rat was given a probe test of the spatial location of the platform after it was removed. The rats' movement tracks were recorded, and the percentages of distance and time spent in the target quadrant were calculated (49).

### Elevated Plus Maze (EPM) Test

In the EPM tests, each rat was placed on the central platform of the EPM with its head pointing in the same direction. After 10 s of adaptation, the rats' movement tracks were recorded. The test box was cleaned between each test using cotton balls containing alcohol to eliminate the impact of the previous experiment. The distance, number of entries to the open or closed arms, and time spent in the open and closed arms were quantified (50).

### Acquisition of MR Images

All MRI experiments were conducted in a 7.0-T horizontal-bore animal scanner (Bruker BioSpec USR 70/30, Bruker BioSpin GmbH, Germany) equipped with actively shielded gradient systems that had a maximum strength of 660 mT/m. Under anesthesia (intraperitoneal injection of 10 mg/kg xylazine and 80 mg/kg ketamine), rats were placed in a prone position on the animal bed and then slid into the center of the magnet bore. Radiofrequency excitation was accomplished with a linear birdcage coil, and a 30-mm receiver surface coil was used for signal reception. A first series of sagittal images was obtained with a turbo spin-echo (TSE) sequence: field of view (FOV) = 35 × 35 mm, matrix size = 240 × 320, slice thickness = 0.69 mm, time echo (TE) = 41 ms, time repetition (TR) = 2,360 ms, number of excitations (NEX) = 2. Then, coronal T2W images were also acquired with a TSE sequence (TR = 3,300 ms, TE = 41 ms, slice thickness = 0.5 mm, FOV = 40 × 40 mm, matrix size = 240 × 320, NEX = 2). After MRI scanning was completed, the rats were perfusion fixed, and their brains were removed for histology.

### Image Selection and Region of Interest (ROI) Delineation

**Figure 1:**
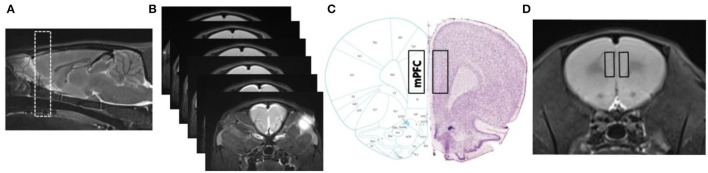
Spatial registration between MR images and histology (Paxinos and Watson, The Rat Brain in Stereotaxic Coordinates, 6th edition). **(A,B)** Sagittal T2W images and consecutive coronal T2W images of mPFC. Detailed range (rectangles) in mPFC slices and MR images considered for immunofluorescence quantification and texture extraction, as shown in **(C,D)**.

The MR image quality was evaluated, and the slices for feature extraction were selected by two radiologists (SZ and JC). The sagittal T2W images and rat-brain stereotaxic coordinates were used as anatomical references for the ROI delineation and placement, and the coronal T2W images were used for feature extraction (Figures 1A,B). For further histological studies, a visual coarse spatial registration was also carried out to define the correspondences between the MR images and the histological slices (Figures 1C,D).

Delineation of ROIs and texture extraction were performed by using the MaZda v4.6 software package (Technical University of Lodz, Poland). To minimize confounding effects, images were normalized by discarding image intensities not within μ ± 3SD (μ: gray-level mean, SD: gray-level standard deviation) (51). To ensure the accuracy and stability of texture extraction, ROIs were delineated as rectangles with the same size (8 × 9) and within the detailed region of the mPFC. Ten ROIs were extracted from the mPFCs recovered from five coronal brain slices from each rat (two ROIs on the left and right mPFC were pooled in each slice). The ROIs were manually placed by radiologists SZ and JC, and radiologist SZ placed the ROIs again within a 2-week interval.

### MRI Texture Extraction and Analysis

A total of 262 texture features were extracted from each ROI using MaZda, and the results were saved as “.sel” files. In this study, texture features were classified into the following major categories: gray-level histogram, gray-level co-occurrence matrix (GLCM), gray absolute gradient matrices, gray run-length matrix, and autoregressive model. Stepwise discriminant analysis (SDA) was used to identify which texture parameters could characterize the changes of microstructure in mPFC ROIs among the five groups. The SDA considered the parameter(s) that were selected in the previous steps to obtain the best classification using linear classification functions. The parameters that have much higher coefficients than the others will show in these functions, and the ability of these parameters to ensure an acceptable classification was verified. The value of this texture parameter in each ROI could then be used as an evaluation of the microstructural changes (52). A least absolute shrinkage and selection operator (LASSO) regression, which is a sparse learning method, was used to select optimal features from high-dimensional data and build radiomics signatures (53). Briefly, LASSO regression reduced the penalty term lambda, set the coefficient of diagnostic-unrelated features to zero, and retained optimal features with non-zero coefficients. After lambda was determined, the radiomics signature was generated by multivariable LASSO regression analysis. Specifically, the radiomics signature was a linear equation composed of intercept and optimal features multiplied by their respective coefficients. For each ROI in the control and SPS groups, we substituted the feature values into the equation to obtain their radiomics score and thus classify them.

### Perfusion-Based Sections

Under deep anesthesia, rats were transcardially perfused with 4% paraformaldehyde in phosphate-buffered saline (PBS) through the left ventricle. The whole brains were removed and immersed in 30% sucrose solution at 4°C and then frozen in liquid nitrogen for cryosections. Then, 15-μm thickness serial frontal sections of brain tissue were sectioned using a cryostat microtome (CM3050 S, Leica Biosystems Nussloch GmbH, Nussloch, Germany) and stored at −20°C in preparation for immunofluorescence staining.

### Immunofluorescence Staining

After being washed with PBS, sections were treated with 5 % bovine serum albumin and 0.3% Triton X-100 in PBS for 30 min at 24°C, followed by washing with PBS. Sections were incubated with mouse anti-glial fibrillary acidic protein (GFAP) polyclonal antibody (Santa Cruz Biotechnology, USA; 1:500) or mouse anti-neuronal nuclei (NeuN) polyclonal antibody (Abcam PLC, UK; 1:1000) at 4°C overnight, respectively. After washing with PBS, sections were incubated with Cy3-conjugated secondary antibody (Boster Biotechnology, China; 1:200) for 2 h at 24°C and re-washed with PBS. Subsequently, the sections were stained with 4′,6-diamidino-2-phenylindole (DAPI; Boster Biotechnology, China) at 37°C for 20 min in the dark and re-washed with PBS, and they were then observed using a fluorescence confocal microscope (Eclipse80i, Nikon, Tokyo, Japan). Three slides were randomly selected from each rat; from each, two visual fields in the bilateral mPFC were randomly selected. The fluorescence intensity and number of positive cells were analyzed using MetaMorph/DPIO/BX41 morphology image analysis system.

### Statistical Analysis

All statistical analyses were performed using SPSS v22.0 (IBM, Armonk, NY, USA), RStudio v3.4.3 (R Foundation for Statistical Computing, Vienna, Austria), and GraphPad Prism v8.0 (GraphPad Software, Inc., San Diego, CA). Data are expressed as mean ± SD. The differences in behavioral tests and immunofluorescence between the control and SPS groups were investigated by performing Student's *t*-tests and one-way analysis of variance (ANOVA) with Tukey's honest significant difference *post-hoc* test for multiple comparisons. Inter- and intra-observer agreements of feature extraction reproducibility were evaluated using inter- and intra-class correlation coefficients (ICCs). SDA was used to identify which texture parameter(s) gave the best classification, and this yielded linear classification functions. LASSO regression was performed using the “glmnet” R package and screen classification of optimal features in the control and each SPS group. Receiver operating characteristic (ROC) curves were used to evaluate the classification performance of the radiomics signatures. The goodness-of-fit of the radiomics signatures was evaluated via the Hosmer–Lemeshow test. A level of *P* < 0.05 was considered to be statistically significant.

## Results

### MWM and EPM Test Results

**Figure 2:**
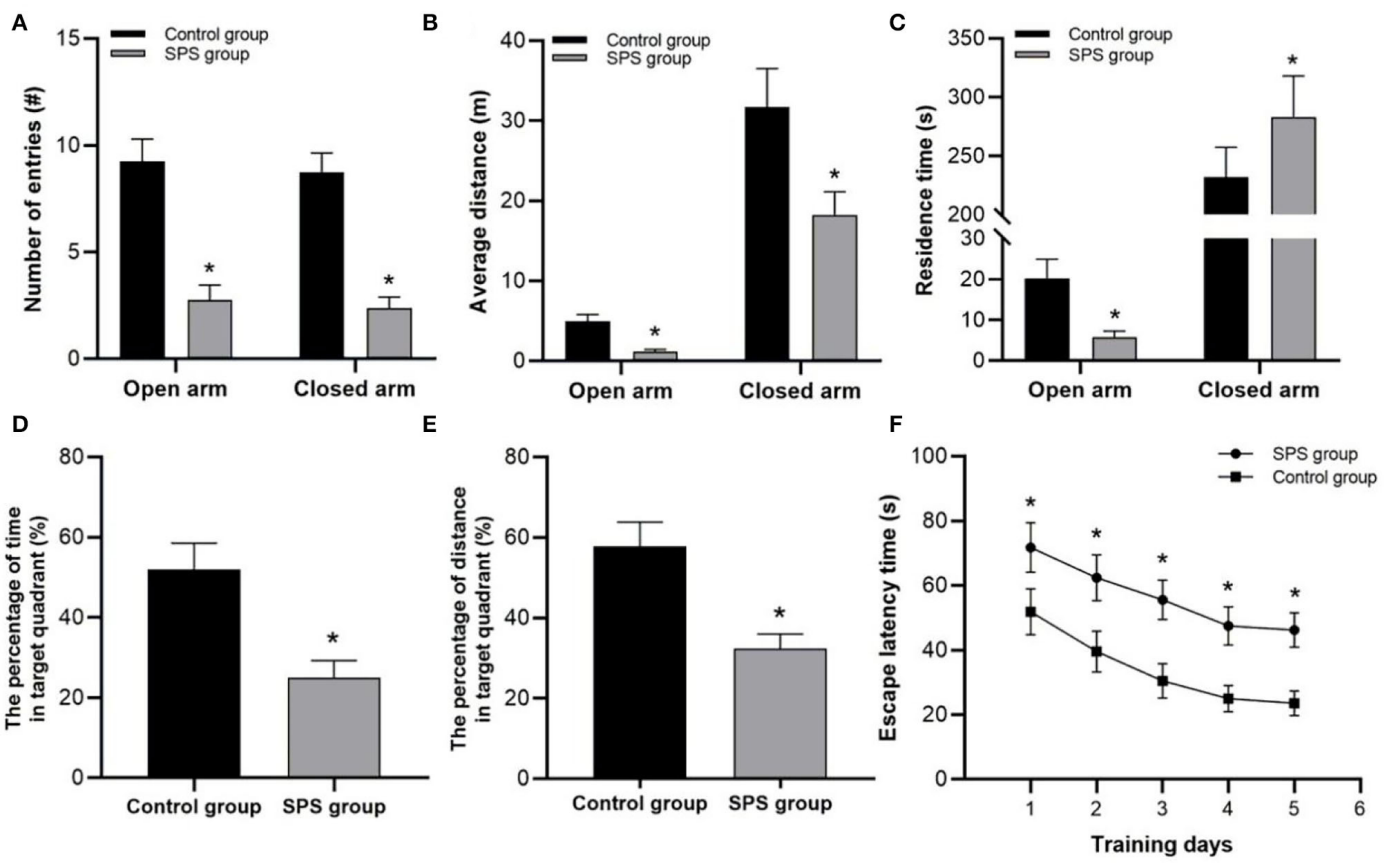
Behavioral test results. **(A)** Number of entries into open and closed arms. **(B)** Average distance traveled in open and closed arms. **(C)** Residence time in open and closed arms. **(D)** Percentage of swimming time in target quadrant. **(E)** Percentage of swimming distance in target quadrant. **(F)** ELT in 5 test days. *P < 0.05 vs. control group.

In the EPM tests, the SPS rats exhibited fewer entries into both the open and closed arms, and they had shorter distance traveled in both the open and closed arms than the control rats (Figures 2A,B). Compared with the control rats, the SPS rats showed lower residence times in the open arms and higher residence times in the closed arms (Figure 2C). In the MWM tests, the SPS rats showed a significant increase in ELTs compared with the control rats from day 1 to day 5 (Figure 2F). The spatial memory tests showed that the SPS rats spent a much lower percentage of time and had a lower percentage of distance traveled in the target quadrant compared with control rats (Figures 2D,E). These behavioral results indicated decreased learning and spatial-memory performance, as well as increased anxiety-like behavior, after SPS stimulation.

### Texture-Feature Extraction and Analysis

**Figure 3:**
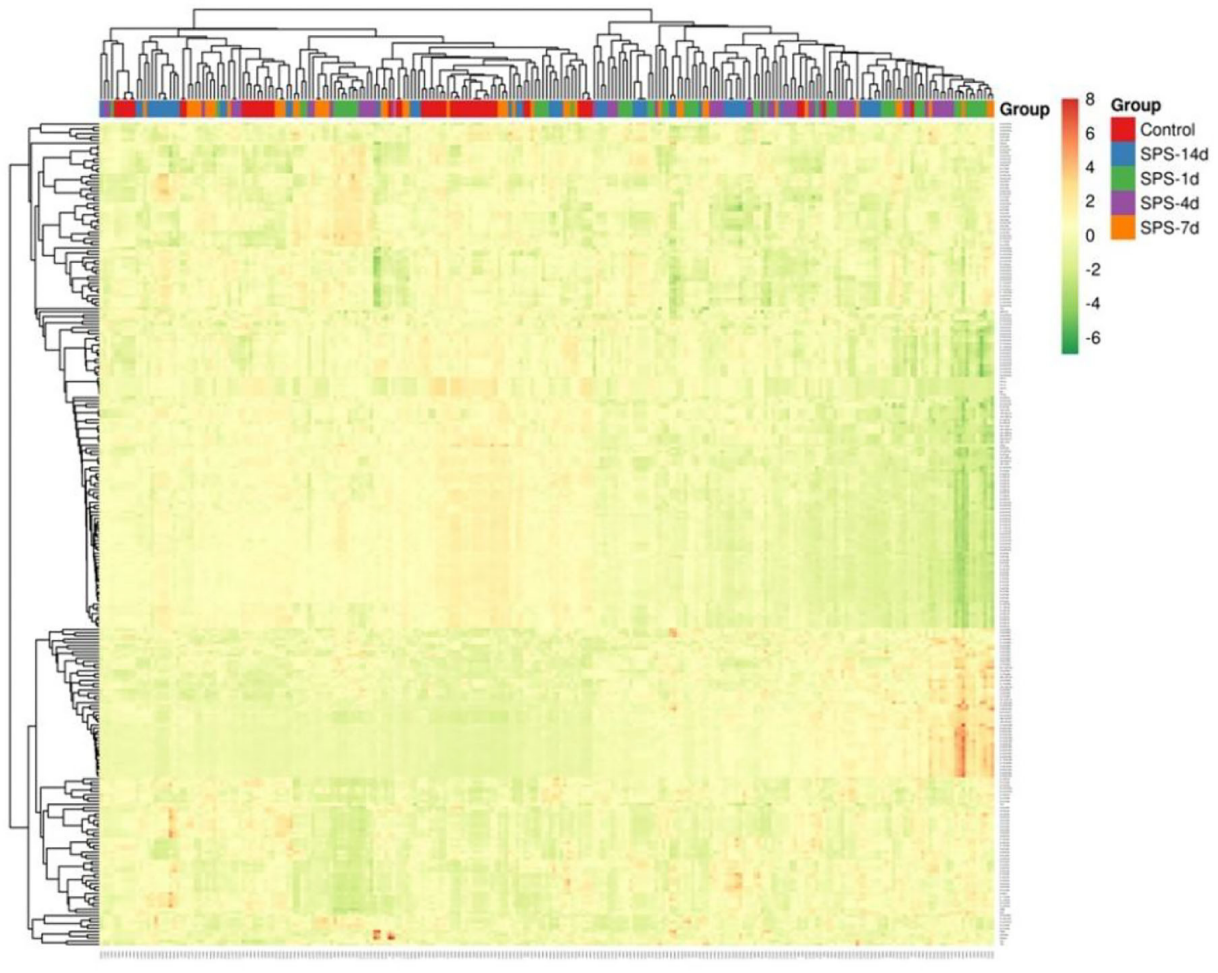
Visualization of radiomics based on T2W images in mPFC. Cluster map of T2W-image radiomics in mPFC, with 245 ROIs (samples) from control and SPS groups on the x axis and 262 radiomic features on the y axis. Each feature was normalized to zero mean and unit standard variance. The ROIs of a given cluster (adjacent columns) shared similar radiomic features in correlation distance.

The rats in the control and each of the SPS groups survived until the end of observation period. The numbers of rats successfully imaged on each MRI examination date and the numbers of sampled ROIs are shown in Table 1. A total of 64,190 texture features were extracted from 245 ROIs. Figure 3 reveals clusters of rats in a specific group with similar radiomic expression patterns based on the T2W images [cluster map visualized via ClustVis (54)].

**Table 1:** Numbers of rats examined by MRI and the numbers of sampled ROIs.

	**Control**	**SPS 1d**	**SPS 4d**	**SPS 7d**	**SPS 14d**
Numbers of rats	10	10	10	9	10
Numbers of ROIs	50	50	50	45	50

After the initial texture features were extracted, the inter- and intra-observer agreements of feature-extraction reproducibility were evaluated by ICCs. The inter- and intra-observer ICC values were calculated using each texture feature for agreement between two radiologists (SZ and JC) and two performances by one radiologist (SZ), respectively. The inter- and intra-observer correlation coefficients ranged from 0.807 to 0.929 and 0.826 to 0.938, respectively, which demonstrates satisfactory reproducibility of feature extraction.

### SDA Results

Firstly, SDA yielded four canonical discriminant functions corresponding to five groups. Each function is a linear combination of the texture parameters that yielded the best discrimination. For a given ROI, a classification score could be calculated using the classification functions, and then it could be classified into a specific group. We obtained the correct classifications with the following features: *X*_1_ = Perc.10%, *X*_2_ = S(1,0)InvDfMom, *X*_3_ = S(0,1)DifEntrp, *X*_4_ = S(1,−1)Entropy, *X*_5_ = S(2,0)InvDfMom, *X*_6_ = S(2,0)SumAverg, *X*_7_ = S(3,0)AngScMom, *X*_8_ = S(3,3)Correlat, *X*_9_ = S(3,3)DifVarnc, *X*_10_ = S(4,0)Contrast, *X*_11_ = S(4,0)InvDfMom, *X*_12_ = S(4,0)DifVarnc, *X*_13_ = S(0,4)DifEntrp, *X*_14_ = S(4,4)AngScMom, *X*_15_ = S(4,-4)SumEntrp, *X*_16_ = S(5,0)Correlat, *X*_17_ = S(5,0)Entropy, *X*_18_ = S(0,5)InvDfMom, *X*_19_ = S(5,5)AngScMom, *X*_20_ = S(5,5)SumEntrp, and *X*_21_ = Teta3. The eigenvalues of the four canonical discriminant functions were 2.178, 1.538, 0.838, and 0.518; their contributions (% of variance) were 42.9, 30.3, 16.5, and 10.2; their Wilks'lambda values were 0.044, 1.141, 0.358, and 0.659 (χ^2^ = 719.38, 452.31, 237.13, and 96.49; *P* all <0.05), respectively. To further discriminate the control and each SPS group, the following five Fisher linear discriminant functions were established:


$$\begin{array}{l} Y_{1}=-2292.719+0.949X_{1}+824.352X_{2}-135.774X_{3}\\ \;+509.869X_{4}+92.995X_{5}\;+19.055X_{6}+12704.031X_{7}\\ \;-92.374X_{8}-0.084X_{9}+1.163X_{10}+255.818X_{11}\\ \;-1.985X_{12}+54.630X_{13}+1433.680X_{14}+32.173X_{15}\\ \;+5.226X_{16}+647.747X_{17}\;+430.129X_{18}+2611.453X_{19}\\ \;+254.037X_{20}+\;36.736X_{21},\\ Y_{2}=-2247.184+0.829X_{1}+835.211X_{2}-154.710X_{3}\\ \;+473.339X_{4}+67.728X_{5}\;+19.338X_{6}+12031.888X_{7}\\ \;-80.063X_{8}+0.008X_{9}+1.153X_{10}+294.170X_{11}\\ \;-1.984X_{12}+60.536X_{13}+1858.346X_{14}+23.455X_{15}\\ \;+4.196X_{16}+679.793X_{17}\;+427.754X_{18}+2427.192X_{19}\\ \;+245.623X_{20}+\;33.608X_{21},\\ Y_{3}=-2156.604+0.749X_{1}+804.413X_{2}-148.971X_{3}\\ \;+476.936X_{4}+80.657X_{5}\;+19.308X_{6}+11934.474X_{7}\\ \;-83.126X_{8}-0.014X_{9}+1.062X_{10}+288.070X_{11}\\ \;-1.859X_{12}+40.440X_{13}+1677.316X_{14}+32.639X_{15}\\ \;-3.429X_{16}+650.782X_{17}\;+395.441X_{18}+2407.338X_{19}\\ \;+248.701X_{20}+\;35.821X_{21},\\ Y_{4}=-2224.278+0.583X_{1}+781.072X_{2}-126.008X_{3}\\ \;+508.944X_{4}+123.048X_{5}\;+19.835X_{6}+12571.383X_{7}\\ \;-88.450X_{8}-0.096X_{9}+1.141X_{10}+270.227X_{11}\\ \;-1.872X_{12}+46.123X_{13}+1338.626X_{14}+25.774X_{15}\\ \;+10.086X_{16}+639.100X_{17}\;+404.116X_{18}+2592.253X_{19}\\ \;+245.984X_{20}+\;24.784X_{21},\\ Y_{5}=-2186.556+0.716X_{1}+769.323X_{2}-134.100X_{3}\\ \;+485.945X_{4}+84.945X_{5}\;+19.815X_{6}+12270.521X_{7}\\ \;-82.620X_{8}-0.089X_{9}+1.122X_{10}+281.944X_{11}\\ \;-1.861X_{12}+73.707X_{13}+1540.184X_{14}+11.549X_{15}\\ \;+2.981X_{16}+638.312X_{17}\;+417.143X_{18}+2475.405X_{19}\\ \;+234.816X_{20}+\;31.604X_{21}. \end{array}$$

Secondly, the previous classification process was used as a basis for prediction concerning the 245 ROIs from 49 rats in the control and each SPS group that we wanted to classify. The rates of correct classification were 92.0, 82.0, 84.0, 86.7, and 88.0% (non-cross validated), and 84.0, 70.0, 84.0, 80.0, and 84.0% (cross validated), respectively, in each group. The general discrimination accuracies of the non-cross-validated and cross-validated functions were 86.5 and 80.4%, respectively. These results indicate that the SPS stimulation yielded a modification of the structure parameters in the mPFC, and these textures features can reflect the characteristics of each group and classify the control or SPS rats.

### LASSO Regression Results

**Figure 4:**
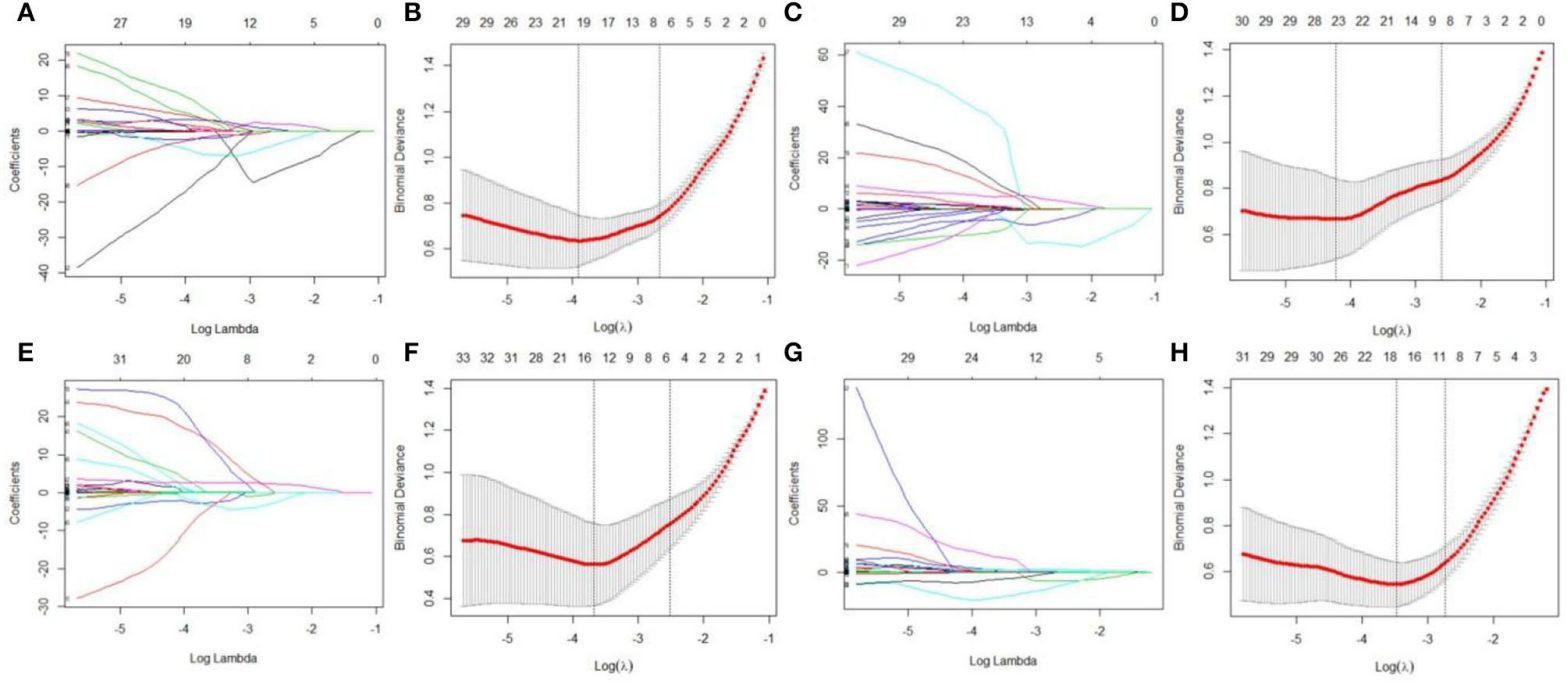
Dimensionality reduction of features with LASSO regression between control and each SPS group. Each colored line represent coefficients of texture features, which are plotted vs. ln(lambda) in the mPFC radiomics signatures between the control and each SPS group: **(A)** control vs. SPS 1d; **(C)** control vs. SPS 4d; **(E)** control vs. SPS 7d; **(G)** control vs. SPS 14d. The binomial deviances are plotted vs. ln(lambda) in mPFC radiomics signatures between control and each SPS group: **(B)** control vs. SPS 1d; **(D)** control vs. SPS 4d; **(F)** control vs. SPS 7d; **(H)** control vs. SPS 14d. Red points indicate average values of deviance for each lambda; two dotted vertical lines correspond to lambda in minimum criteria and one standard error of minimum criteria.

**Figure 5:**
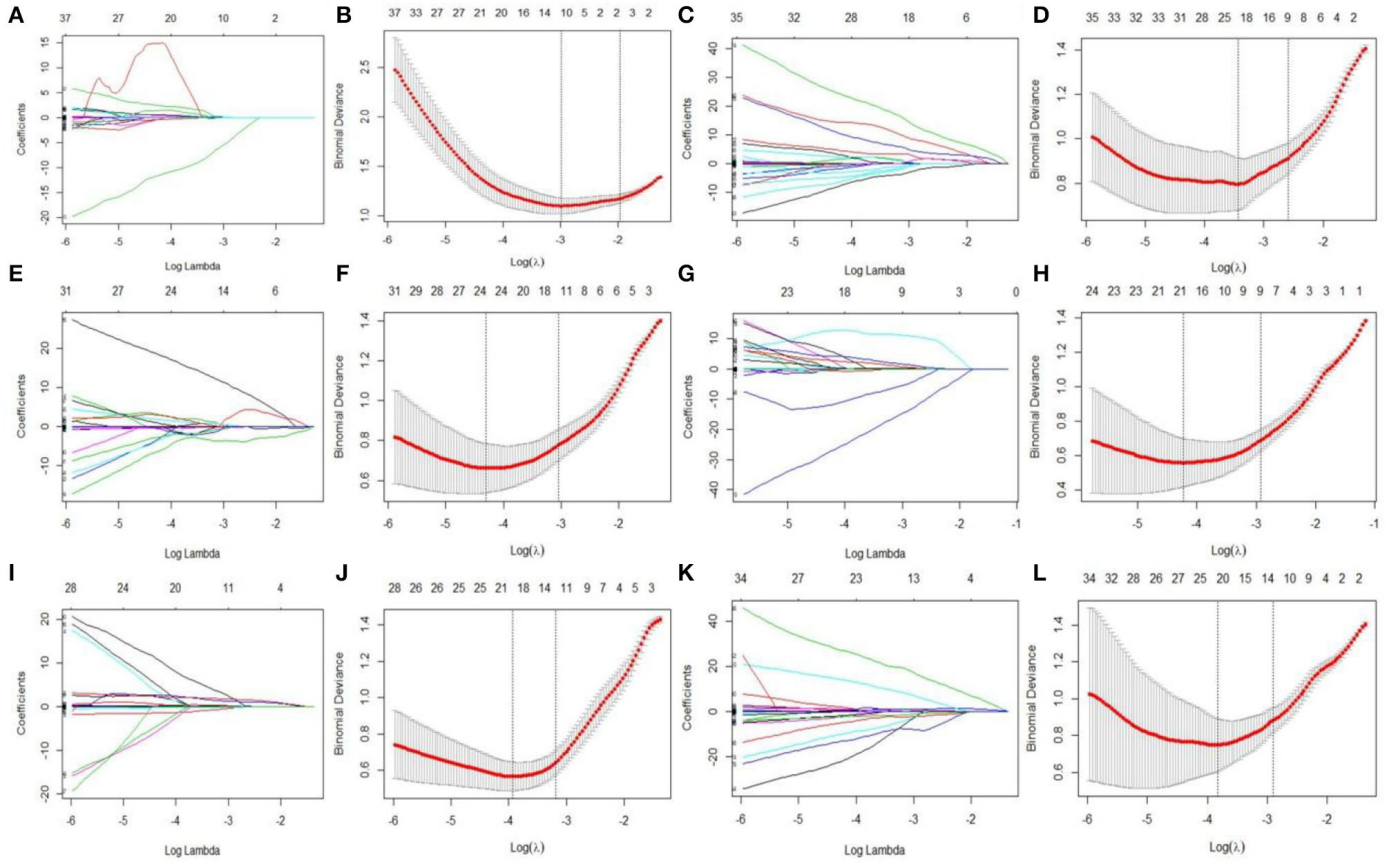
Features dimensionality reduction with LASSO regression among four SPS groups. Each colored line represent coefficients of texture features, which plot vs. ln(lambda) in mPFC radiomics signatures between each SPS groups: **(A)** SPS 1d vs. SPS 4d; **(C)** SPS 1d vs. SPS 7d; **(E)** SPS 1d vs. SPS 14d; **(G)** SPS 4d vs. SPS 7d; **(I)** SPS 4d vs. SPS 14d; **(K)** SPS 7d vs. SPS 14d. The binomial deviances were plotted vs. ln(lambda) in mPFC radiomics signatures between each SPS groups: **(B)** SPS 1d vs. SPS 4d; **(D)** SPS 1d vs. SPS 7d; **(F)** SPS 1d vs. SPS 14d; **(H)** SPS 4d vs. SPS 7d; **(J)** SPS 4d vs. SPS 14d; **(L)** SPS 7d vs. SPS 14d.

LASSO regression model with 10-fold cross-validation was employed to select predictive features among the preliminarily extracted texture parameters (37). Figure 4 shows trace plots of the texture feature coefficients fit by LASSO and determination of the penalty term lambda. The optimized lambda values for the left dotted vertical lines were selected to determine the minimization criteria of binomial deviance. Lambda values of 0.020 [ln(lambda) = −3.912], 0.015 [ln(lambda) = −4.200], 0.025 [ln(lambda) = −3.689], and 0.031 [ln(lambda) = −3.474] were selected for the mPFC radiomics signatures between the control and each SPS group, respectively. After dimensionality reduction, there were 21, 23, 14, and 17 optimal features with non-zero coefficients remaining in the mPFC radiomics signatures between the control and each SPS group (Table 2). Among four SPS groups, Lambda values of 0.050 [ln(lambda) = −2.996], 0.033 [ln(lambda) = −3.411], 0.013 [ln(lambda) = −4.343], 0.014 [ln(lambda) = −4.269], 0.019 [ln(lambda) = −3.963], and 0.022 [ln(lambda) = −3.817] were selected (Figure 5). Six classification models containing 10, 18, 27, 21, 20, and 21 features were established after LASSO dimensionality reduction between SPS 1d and SPS 4d, SPS 1d and SPS 7d, SPS 1d and SPS 14d, SPS 4d and SPS 7d, SPS 4d and SPS 14d, SPS 7d and SPS 14d, respectively (Table 3).

**Table 2:** Optimal mPFC features after dimensionality reduction between the control and each SPS group via LASSO regression.

**Group**	**Intercept**	**Optimal Features (Coefficient)**
Control vs. SPS 1d	−26.323	S(1,0)SumAverg (−0.113); S(1,0)SumEntrp (0.678); S(0,1)SumEntrp (0.423); S(2,0)InvDfMom (4.703); S(0,2)Correlat (−2.241); S(0,2)InvDfMom (−5.431); S(2,2)DifVarnc (−0.008); S(2,-2)Contrast (0.007); S(4,0)InvDfMom (−15.567); S(4,4)InvDfMom (−2.478); S(4,-4)DifEntrp (3.256); S(5,0)Contrast (−0.003); S(5,0)Correlat (0.180); S(5,0)InvDfMom (1.569); S(5,0)SumAverg (0.011); S(0,5)DifVarnc (0.030); S(5,5)SumAverg (0.039); S(5,5)SumEntrp (4.152); S(5,-5)SumVarnc (−0.002); 45dgr_RLNonUni (0.123); GrNonZeros (9.046)
Control vs. SPS 4d	−4.487e+01	S(1,0)SumAverg (−1.795e-01); S(0,1)Correlat (−1.470e+00); S(2,0)InvDfMom (2.181e+01); S(2,0)DifEntrp (2.942e+00); S(0,2)InvDfMom (−4.939e+00); S(0,2)SumVarnc (−4.707e-04); S(2,2)SumOfSqs (−1.966e-02); S(2,2)DifVarnc (−1.453e-02); S(2,-2)SumVarnc (−7.999e-03); S(3,0)InvDfMom (−6.434e+00); S(3,3)DifVarnc (−1.026e-02); S(4,0)InvDfMom (−1.061e+01); S(4,4)InvDfMom (−2.443e+00); S(4,-4)DifEntrp (6.212e+00); S(5,0)Contrast (−6.959e-03); S(0,5)DifVarnc (1.060e-02); S(5,5)SumEntrp (1.823e+00); Horzl_LngREmph (2.518e-02); Vertl_RLNonUni (1.930e-02); 45dgr_Fraction (4.565e+01); 135dr_ShrtREmp (−1.150e+01); GrMean (1.516e+00); GrNonZeros (1.511e+01)
Control vs. SPS 7d	−48.294	MinNorm (0.104); Kurtosis (0.153); S(1,1)InvDfMom (−2.713); S(1,1)DifVarnc (0.085); S(1,-1)Contrast (−0.002); S(1,-1)InvDfMom (14.507); S(3,-3)InvDfMom (0.354); S(4,0)InvDfMom (−2.992); S(5,0)InvDfMom(−4.858); S(5,0)SumAverg (0.201); S(0,5)DifVarnc (0.003); S(5,5)DifVarnc (0.002); GrNonZeros (14.086); Teta3 (2.679)
Control vs. SPS 14d	−2.511e+01	MinNorm (8.246e-02); S(2,0)InvDfMom (3.396e-02); S(2,0)SumAverg (−4.167e-02); S(0,2)InvDfMom (−5.022e+00); S(2,2)SumVarnc (−4.633e-03); S(2,-2)SumAverg (−3.943e-03); S(3,-3)SumAverg (−4.006e-02); S(4,0)InvDfMom (−1.827e+01); S(4,0)DifVarnc (−5.782e-03); S(5,0)Correlat (1.598e+00); S(5,0)SumAverg (1.341e-01); S(5,0)SumVarnc (1.653e-03); S(5,0)DifVarnc (−7.598e-03); S(5,5)Contrast (−2.789e-04); S(5,5)DifVarnc (−2.237e-03); Horzl_LngREmph (1.750e+00); 45dgr_ShrtREmp (1.058e+01)

**Table 3:** Optimal mPFC features after dimensionality reduction among four SPS groups via LASSO regression.

**Group**	**Intercept**	**Optimal Features (Coefficient)**
SPS 1d vs. SPS 4d	−1.177e+01	MinNorm (6.278e-02); S(2,0)SumOfSqs (9.153e-03); S(2,-2)SumOfSqs (1.334e-02); S(3,-3)Correlat (1.802e-01); S(3,-3)SumVarnc (6.736e-04); S(4,-4)Contrast (−4.929e-04); S(5,0)SumOfSqs (−2.089e-03); S(5,0)InvDfMom (−5.440e+00); S(5,0)DifVarnc (−2.695e-04); GrSkewness (5.052e-02)
SPS 1d vs. SPS 7d	−9.741	S(1,0)DifEntrp (−0.121); S(1,1)SumVarnc (0.001); S(1,-1)Correlat (1.926); S(2,0)InvDfMom (−2.477); S(2,0)DifVarnc (−0.027); S(2,2)Correlat (0.179); S(2,2)InvDfMom (12.293); S(0,3)InvDfMom (6.702); S(3,3)SumAverg (−0.049); S(0,4)InvDfMom (18.735); S(5,0)Contrast (0.003); S(5,0)InvDfMom (−1.416); S(0,5)InvDfMom (3.210); S(0,5)DifVarnc (−0.004); S(5,-5)InvDfMom (1.655); S(5,-5)SumAverg (0.038); Teta2 (−3.010); Teta4 (−4.267)
SPS 1d vs. SPS 14d	23.514	Skewness (−0.450); S(1,0)DifEntrp (−11.303); S(1,1)InvDfMom (−7.960); S(1,-1)InvDfMom (−7.592); S(2,0)SumAverg (−0.484); S(0,2)DifVarnc (−0.083); S(2,2)InvDfMom (2.953); S(2,2)DifVarnc (0.013); S(0,3)SumOfSqs (−0.025); S(0,3)InvDfMom (22.303); S(3,3)DifVarnc (0.013); S(4,0)DifVarnc (−0.001); S(0,4)DifVarnc (−0.029); S(4,4)Entropy (−2.369); S(5,0)SumVarnc (0.027); S(5,0)Entropy (3.405); S(0,5)DifVarnc (−0.014); S(5,5)Contrast (−0.007); S(5,5)SumVarnc (0.003); S(5,5)SumEntrp (3.423); S(5,5)DifVarnc (0.016); S(5,-5)SumOfSqs (0.004); S(5,-5)DifVarnc (0.002); Horzl_LngREmph (3.061); GrVariance (−0.156); Teta1 (2.438); Teta2 (−5.445)
SPS 4d vs. SPS 7d	−26.622	MinNorm (0.132); Perc.01% (0.028); S(0,1)InvDfMom (0.524); S(1,-1)Correlat (0.525); S(1,-1)SumVarnc (0.008); S(1,-1)DifEntrp (−0.297); S(0,2)InvDfMom (12.687); S(2,2)InvDfMom (2.753); S(0,3)InvDfMom (0.302); S(4,4)SumEntrp (−0.536); S(4,4)DifVarnc (0.019); S(5,0)InvDfMom (−27.167); S(5,0)SumAverg (0.079); S(5,5)DifVarnc (0.001); S(5,-5)InvDfMom (2.816); Horzl_ShrtREmp (−11.808); 45dgr_RLNonUni (−0.073); GrKurtosis (−0.236); GrNonZeros (5.767); Teta2 (−0.274); Teta3 (4.306)
SPS 4d vs. SPS 14d	−7.730	MinNorm (0.142); Skewness (−1.296); S(2,0)DifEntrp (−1.964); S(2,2)InvDfMom (7.538); S(2,2)DifVarnc (0.007); S(2,-2)Correlat (2.366); S(2,-2)SumAverg (−0.127); S(0,3)InvDfMom (1.492); S(3,3)DifVarnc (0.001); S(3,-3)SumAverg (−0.190); S(4,-4)Correlat (0.416); S(4,-4)SumEntrp (1.088); S(5,0)InvDfMom (−1.220); S(5,0)Entropy (0.953); S(5,0)DifVarnc (−0.026); S(0,5)Contrast (−0.005); S(0,5)Correlat (0.299); S(0,5)DifVarnc (−0.001); S(5,5)SumVarnc (0.002); Horzl_LngREmph (2.157)
SPS 7d vs. SPS 14d	2.020e+01	S(1,0)InvDfMom (1.655e+00); S(1,1)Contrast (−1.581e-03); S(1,-1)InvDfMom (−1.715e+01); S(2,0)SumOfSqs (−1.771e-02); S(2,0)InvDfMom (2.394e+01); S(0,2)InvDfMom (−1.110e+01); S(2,2)SumOfSqs (−8.780e-03); S(3,0)DifVarnc (9.913e-03); S(3,3)InvDfMom (−3.492e+00); S(0,4)InvDfMom (−8.386e+00); S(4,-4)Correlat (9.364e-03); S(4,-4)SumVarnc (9.205e-04); S(5,0)Correlat (6.560e-01); S(5,0)InvDfMom (1.203e+01); S(5,0)SumVarnc (3.034e-03); S(0,5)DifVarnc (−4.283e-02); S(5,5)Entropy (−1.489e+00); S(5,5)DifEntrp (−9.448e-01); Horzl_LngREmph (6.066e-01); Teta1 (1.758e-01); Teta3 (−2.076e+00)

Plots of ROC curves were used to evaluate the performance of the mPFC radiomics signatures for classifying PTSD rats among control and SPS groups. The area under the ROC curve (AUC) values were 0.944 [95% confidence interval (CI): 0.903–0.984], 0.950 (95% CI: 0.914–0.987), 0.959 (95% CI: 0.923–0.996), and 0.936 (95% CI: 0.891–0.980) between the control and the four SPS groups, respectively. The AUC values were 0.927 [95% CI: 0.882–0.973], 0.943 [95% CI: 0.885–1.000], 0.967 [95% CI: 0.942–0.996], 0.916 [95% CI: 0.864–0.968], 0.932 [95% CI: 0.889–0.976], and 0.893 [95% CI: 0.822–0.965] between SPS 1d and SPS 4d, SPS 1d and SPS 7d, SPS 1d and SPS 14d, SPS 4d and SPS 7d, SPS 4d and SPS 14d, SPS 7d and SPS 14d, respectively. The Hosmer–Lemeshow test showed acceptable goodness-of-fit of the mPFC radiomics signatures in all of the groups (*P* all > 0.05).

### Cell Nuclear Changes in the mPFC

**Figure 6:**
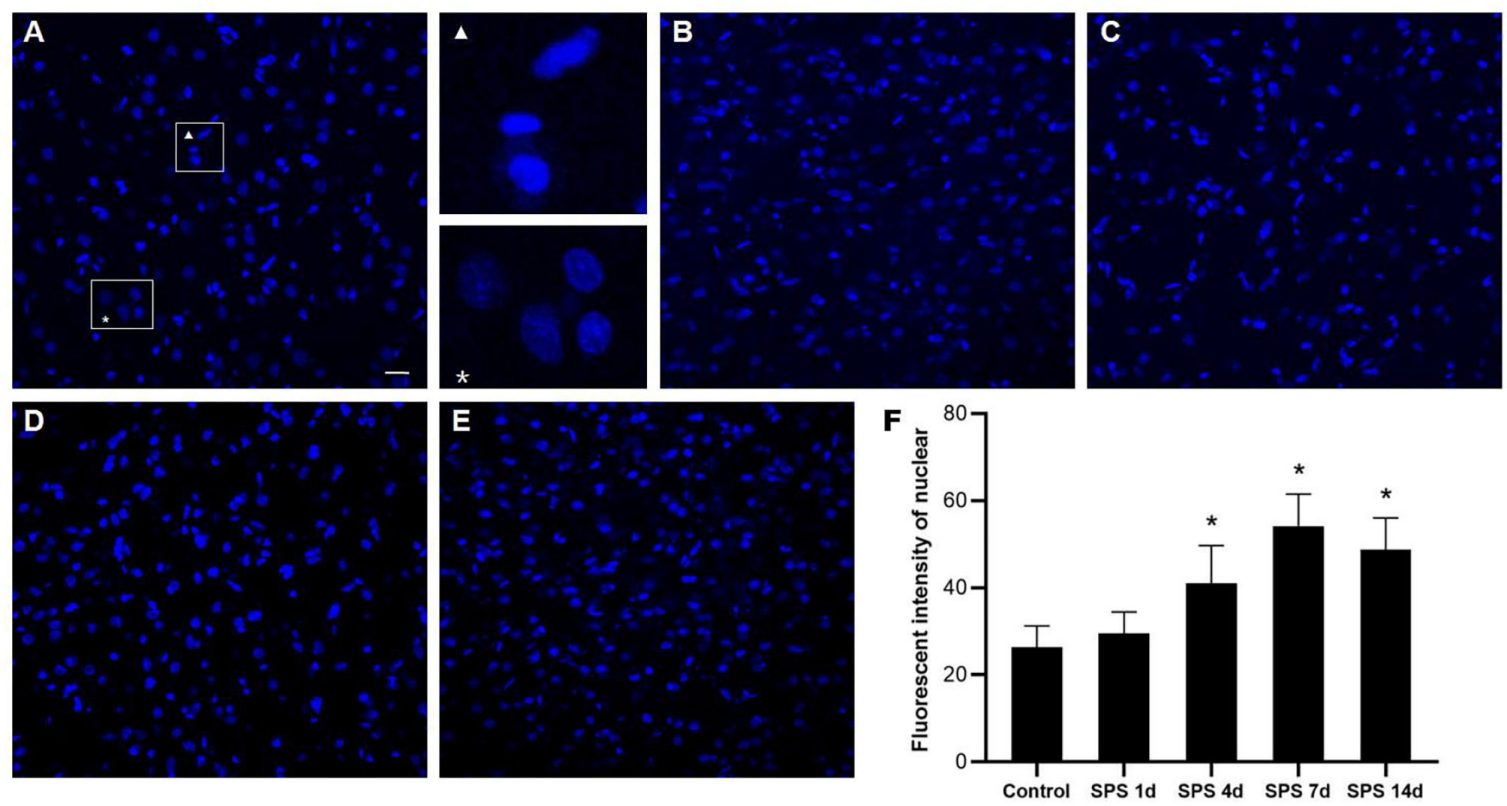
mPFC DAPI immunostaining. Representative photomicrographs of DAPI immunostaining are shown in **(A–E)** (magnification ×400, Bar = 20 μm): **(A)** control group; **(B)** SPS 1d; **(C)** SPS 4d; **(D)** SPS 7d; **(E)** SPS 14d. The normal and abnormal cellular nuclei are shown in the right part of panel **(A)**. Quantitative analysis of fluorescence intensity of cell nuclei in mPFC is shown in panel **(F)**. *P < 0.05 vs. control group.

Morphological changes of the cellular nuclei in the mPFC were detected using DAPI staining. Normal mPFC cellular nuclei showed a relatively dim fluorescence with a little prominent chromatin condensation as well as a diffuse and uniform distribution. After SPS stimulation, numerous mPFC cellular nuclei showed bright blue fluorescence with prominent chromatin condensation and fragmentation, and there were significant increases on days 4 and 7 after SPS stimulation, as shown in Figure 6. The magnified images show normal cellular nuclei (right part of Figure 6A, asterisk) and abnormal cellular nuclei (right part of Figure 6A, triangle), respectively.

### Immunofluorescence of NeuN and GFAP in the mPFC

**Figure 7:**
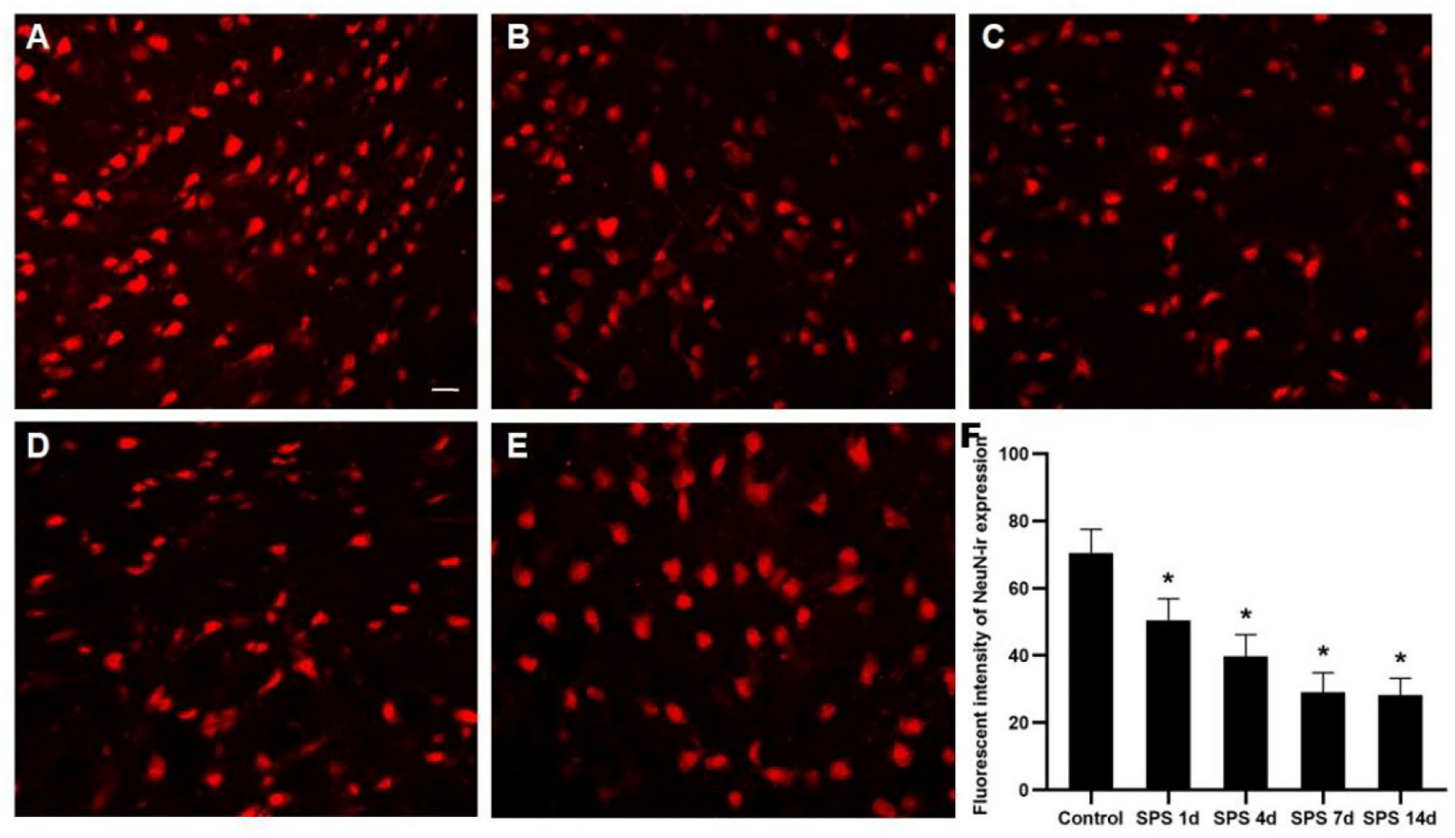
Immunofluorescence observations of GFAP in mPFC. Representative images of GFAP immunoreactivity (IR) and results analysis in mPFC (magnification ×400, Bar = 20 μm): **(A)** control group; **(B)** SPS 1d; **(C)** SPS 4d; **(D)** SPS 7d; **(E)** SPS 14d. **(F)** Quantity analysis of GFAP-IR expression in mPFC in each group. *P < 0.05 vs. control group.

**Figure 8:**
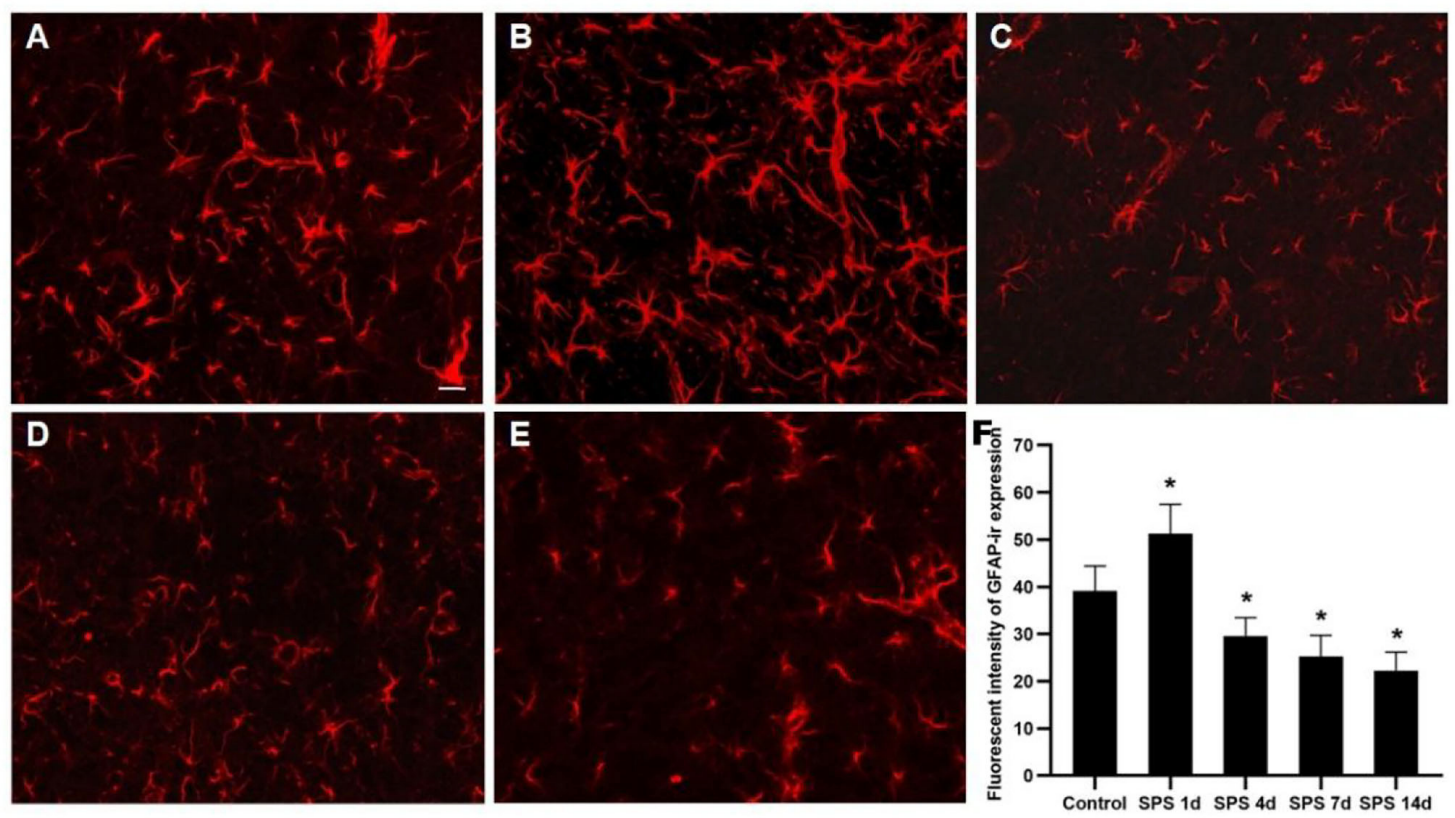
Immunofluorescence observation of NeuN in mPFC. Representative images of NeuN-IR and results analysis in mPFC (magnification ×400, Bar = 20 μm): **(A)** control group; **(B)** SPS 1d; **(C)** SPS 4d; **(D)** SPS 7d; **(E)** SPS 14d. **(F)** Quantity analysis of NeuN-IR expression in mPFC in each group. *P < 0.05 vs. control group.

**Figure 9:**
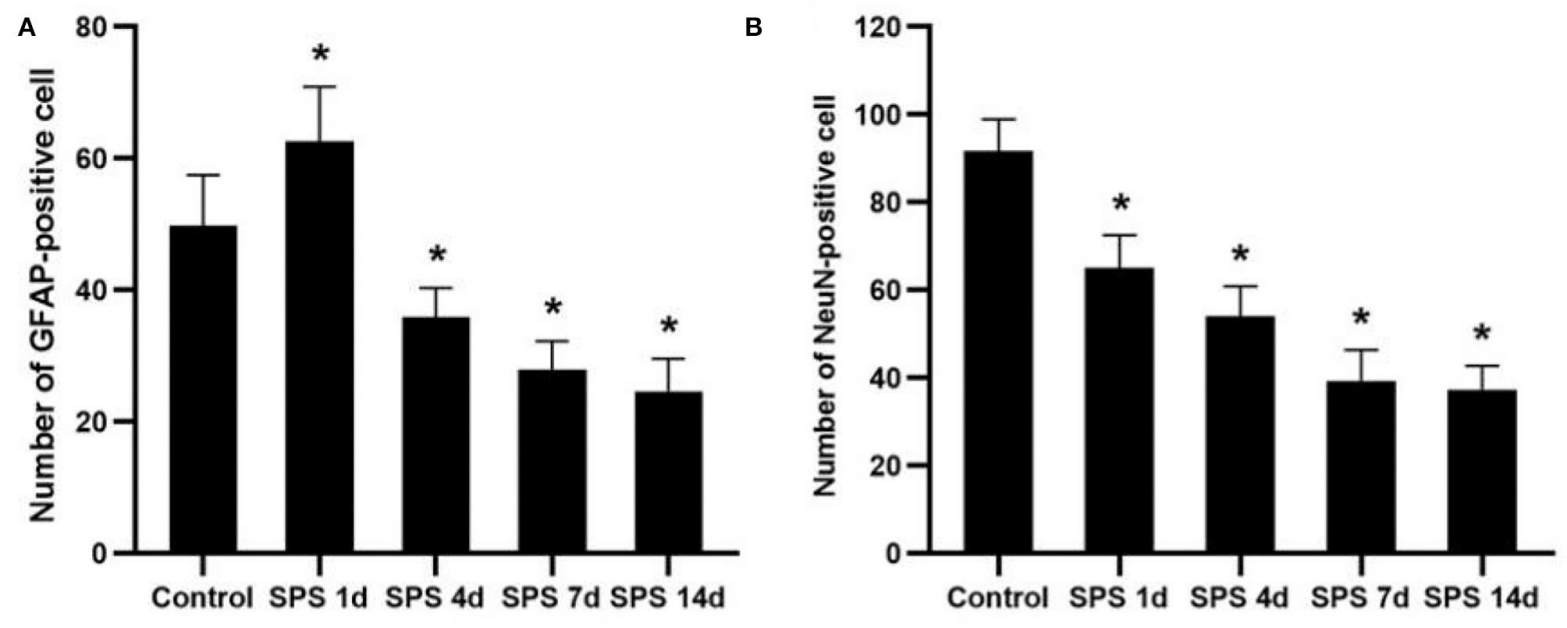
Number of NeuN- and GFAP-positive cells. **(A)** number of GFAP-positive cells in mPFC of control and SPS groups; **(B)** number of NeuN-positive cells in mPFC of control and SPS groups. *P < 0.05 vs. control group.

To evaluate the effects of SPS stimulation on the mPFC neurons and astrocytes, we performed immunofluorescence against NeuN (the specific neuronal marker) and GFAP (the specific astrocyte marker), respectively. Compared with the control group, the fluorescent intensity of GFAP-immunoreactivity (IR) expression increased in the mPFC at 1 day, then decreased 4, 7, and 14 days after SPS stimulation (Figure 7). The fluorescent intensity of NeuN-IR expression decreased from 1 to 14 days after SPS stimulation in comparison with the control group (Figure 8). The same change trends in the number of NeuN- and GFAP-positive cells were also found in the mPFC of each group (Figure 9).

## Discussion

An appropriate animal model is necessary for performing experimental research on PTSD, and this should possess appropriate behavioral and neurobiological characteristics (55). Among various animal models, rat PTSD induced by SPS stimulation is a standard, reproducible and frequently used PTSD model (48). The SPS process mimics the severe stimulation by stressors in humans, and the behavioral endophenotypes and neurobiological alterations [e.g., glucocorticoid receptor hypersensitivity, hypothalamic–pituitary–adrenal (HPA)-axis dysfunction, and abnormal behavior and cognitive performance] in SPS rats correlate well with the clinical manifestations of human PTSD. In previous PTSD animal experiments (concern neurobiological, neuroimaging or neuropathological researches, etc.) (56–59), we found that the period of experiments ranges from hours to days, mostly focused on 1, 4, 7, and 14 days after SPS stimulation. Based on the numerous PTSD pre-clinical studies (18, 60–64), SPS 1d, SPS 4d, SPS 7d, and SPS 14d were designed in this study. A time-dependent sensitization (TDS) study has proposed that the undisturbed period is a necessary condition to produce PTSD-like manifestations (65). Moreover, SPS can lead to enhanced negative feedback of the HPA axis at 7 days after SPS via TDS (66). Thus, behavioral experiments are generally undertaken over a period of 7 days after SPS stimulation. In the present study, the behavioral results demonstrate impaired learning and spatial memory, anxiety-like behaviors, and fear-related avoidance, showing that SPS stimulation successfully induced PTSD in the rat model.

Texture analysis of MR images can reveal and quantify the structural changes appearing at the cellular level, and this provides invisible information regarding the tissues of interest, even using conventional MRI (67). A large number of neurodegenerative diseases such as AD, PD, and amyotrophic lateral sclerosis may be too subtle to be detected by conventional MRI examination. However, MRTA can capture the changes occurring in the early stages of these diseases and could be useful for early diagnosis (67, 68). It has also been reported that non-invasive MRTA can be used as a sensitive and efficient method for detection of cerebral alterations in mice during chronic exposure to glufosinate ammonium (69). To the best of our knowledge, this is the first study to combine non-invasive T2W image MRTA and immunofluorescence methods in the examination of PTSD. We used a general radiomics approach to extract features and establish radiomics models of mPFC that had undergone microstructural changes that were not yet visible on conventional T2W images.

In this study, the optimal features were almost all derived from GLCM categories according to the SDA and the LASSO regression calculation method. As a second-order texture, GLCM takes into account the spatial relationships between pixels and examines second-order statistical information within an image. The subclasses of GLCM are as follows.

(1) Contrast, which represents the degree to which texture intensity levels differ between voxels (local intensity variations), which will favor contributions from P(i,j) away from the diagonal.
(2) AngScMom, which reflects a similar form of physics equations to those used to calculate the angular second moment. AngScMom uses each P(i,j) as a weight (gray distribution uniformity and texture thickness) for itself, and high values of AngScMom occur when the window is very orderly.
(3) InvDfMom, which corresponds to small contributions from inhomogeneous areas (i ≠ j). The value is low for inhomogeneous images and relatively high for homogeneous images.
(4) SumAverg and SumVarnc: SumAverg measures the relationship between occurrences of pairs with lower intensity values and occurrences of pairs with higher intensity values. Quantifying brightness, SumVarnc represents the global variation in the sum of the gray levels of the voxel pair distribution.
(5) SumSqr, also called variance, gives a high weighting to elements that differ from the average value.
(6) Correlation represents the degree of mutual dependency between pixels.
(7) Entropy represents the degree of uncertainty (a measure of randomness).
(8) DifVarnc reflects the texture period: high values indicate a larger texture period.
(9) DifEntrp and SumEntrp relate to the amount of image information. This measures the randomness of image content and indicates the texture complexity (70).

Stepwise methods are frequently employed in educational and psychological research, both to select useful subsets of variables and to evaluate their order of importance (71). Here, the SDA results showed that the correct classification rates were 92.0, 82.0, 84.0, 86.7, and 88.0% (non-cross-validated), and 84.0, 70.0, 84.0, 80.0, and 84.0% (cross-validated) in each group. The general discrimination accuracy of the non-cross-validated and cross-validated groups were 86.5 and 80.4%, respectively. LASSO regression is a popular machine-learning algorithm, and it is widely used as a high-dimensional data-analysis tool in radiomics research. Because LASSO regression is designed to avoid overfitting, it can analyze large sets of texture features with relatively small sample sizes (53). After LASSO dimensionality reduction, four classification models containing 21, 23, 14, and 17 features were established; these achieved AUC values of 0.944, 0.950, 0.959, and 0.936, respectively, for classifying PTSD in the control and each SPS group. Among four SPS groups, six classification models containing 10, 18, 27, 21, 20, and 21 features were established after LASSO dimensionality reduction, respectively. These models achieved AUC values of 0.927, 0.943, 0.967, 0.916, 0.932, and 0.893 for classifying each SPS group. The SDA and LASSO regression results demonstrate that optimal texture features can reflect the mPFC characteristics of each group and can effectively classify control and each SPS rats.

As mentioned above, the mPFC has a crucial role in both PTSD and regulation of fear-memory expression, and structural changes in and dysfunction of the mPFC are important factors leading to memory disorder and fear memory in PTSD patients (39). Our previous studies have shown that SPS stimulation induces enhancement of apoptosis and dysregulation of autophagic activity in mPFC neurons (44, 46). In this study, the fluorescence of cell nuclei gradually increased as a consequence of the pycnotic changes in the mPFC after SPS stimulation, and this was represented by declining presence of cell bodies, cytoplasm concentration, and chromatin. The DAPI fluorescence results demonstrated that SPS increased mPFC neural death. It is generally known that astrocytes perform many functions, including neurotransmitter uptake and inactivation, neuronutrition and repair, and regulation of neuroplasticity (72–74). Many clinical studies have shown that the expression of GFAP is significantly reduced in the brains of patients with generalized depression and depressive disorder (75, 76). Our previous studies have reported that the loss of glial cells causes abnormal hippocampal atrophy and dysfunction in PTSD rats (77, 78). However, it is not clear whether glial cells, neurons, or both are involved in the changes of morphology and function in the mPFC after SPS stimulation. The present study's GFAP-immunofluorescent staining results show that the fluorescence intensity of GFAP-IR expression and number of GFAP-positive cells increased in the mPFC at 1 day and then decreased at 4, 7, and 14 days after SPS stimulation. The number of NeuN-positive cells and intensity of NeuN-IR in each SPS group was significantly lower than that in the control group. These findings indicate dysfunction of the mPFC following neuron and astrocyte loss after SPS stimulation. Changes in astroglia–neuron interactions could also be involved because astrocytes are integral functional elements of the synapses, responding to neuronal activity and regulating synaptic transmission and plasticity (79, 80). The neurobiological evidence from brain imaging and postmortem has demonstrated mPFC alterations at the gross structural and cellular levels in PTSD, as well as the functional consequences of these changes (81). A number of MRI studies have reported that PTSD is characterized by altered size of cortical and limbic brain regions. In PTSD patients, reductions in tissue volume have been found in medial, ventrolateral PFC and hippocampus, and these changes may be attributed, in part, to a reduced number of neurons or glial cell types (81). The findings of present study indicate mPFC neuron and astrocyte loss after SPS stimulation, which is one of the most important reasons in mPFC structural and cellular changes in PTSD patients. Neural death and neuron and astrocyte loss are all associated with microstructural changes in and dysfunction of the mPFC after SPS stimulation, and these are also closely correlated with changes in the texture parameters. MRTA can be used as an efficient method for detection of mPFC microstructural alterations in SPS rats, based on which we speculate that MRI radiomics also could potentially serve as a novel neuroimaging marker in PTSD diagnosis.

In an experimental study by Meme (69), MRTA proved to be an effective and sensitive approach for detection of cerebral alterations in mice following exposure to glufosinate ammonium, a herbicide. This study demonstrated that MRTA can detect changes due to modification of GFAP expression, intracellular glutamate metabolite content, and astrocyte swelling. In our study, we speculate that the changes in texture parameters in the mPFC maybe associated with not only modification of GFAP expression but also with changes of nuclei and NeuN expression after SPS stimulation. Interestingly, we found that the optimal texture parameters were different between the control and each SPS group after dimensionality reduction through LASSO regression, despite the optimal parameters almost all being derived from GLCM categories. The reason for this may be that texture parameters are related not only to the cellular number or ratio (neurons, glial cells, and nuclei) but also to the distribution of cell composition—such as calcium, iron deposition, and water—and molecular content and distribution, in different pixels. All these factors may affect the spatial information and the relevance of pixels, which leads to some differences in optimal texture parameters between each SPS group and the control group after dimensionality reduction. MRTA revealed the comprehensive tissue modifications under the action of various pathological factors in the rat mPFC in response to SPS stimulation. Alterations of neurons, glial cells, and nuclei in the mPFC play important roles in the MRTA changes. Although the optimal texture parameters were different in each subclass, the mPFC radiomics signatures based on these texture parameters had good classification performance both for the control group and the SPS groups.

In AD, PD, Huntington's disease, as well as many other neurological or psychiatric diseases, the diverse pathological characteristics of these disease create a complexity where different exact pathological mechanisms (such as one of the pathological hallmarks of AD is the abnormal aggregation of Aβ peptides, PD is characterized by nigrostriatal degeneration and iron deposition in the neostriatum). MRTA/radiomics pattern can capture the microstructural changes occurring in the early stages of these diseases and could be useful for early diagnosis. However, MRTA/radiomics pattern may not be a specific indicator to a particular disease related pathology in the strict sense. Overall, the present findings show that the mPFC radiomics signatures based on T2W images achieved good classification performance for PTSD rats and showed the potential for non-invasive mPFC radiomic features to be used as a neuroimaging marker for PTSD. Moreover, additional issues need to be addressed in order to refine and further validate the radiomics models; further PTSD clinical studies are therefore warranted.

The present study had several limitations. First, changes in the texture parameters in the mPFC were discussed with a focus on changes in the nucleus, neurons, and astrocytes after SPS stimulation. Other factors, such as the contents of various substances in cells, cell micromorphology (e.g., the length and number of dendrites and the volume of different cells), and even molecular content or distribution, may also be related to the observed changes in texture parameters. This deserves further study. Second, Changes in the texture parameters based on T2W images in the mPFC were analyzed in a PTSD rat model. We should also further explore the texture parameters in other brain regions and multisequence MRI images, and this would establish a radiomics model for diagnosing PTSD in clinical research. Third, SDA and LASSO regression were preliminarily used to reduce the dimensions of texture features and build classification functions or radiomics signatures in the present study, respectively. With the rapid development of artificial intelligence, various artificial neural network and machine-learning algorithms can be further applied in PTSD radiomics research.

## Conclusion

The present study provides evidence that SPS induces nucleus alterations and neuron and glial-cell loss in the mPFC, and these have been shown to be important in microstructural changes and dysfunction within the mPFC. All of these microstructural changes might be characterized by changes in the textural patterns in T2W images, and these can be captured using a radiomics approach. The mPFC radiomics signatures based on T2W images in this study achieved good classification performance for PTSD rats. Our primary results showed the potential for non-invasive mPFC radiomic features to be used as a novel neuroimaging marker for PTSD, and this could potentially serve as a basis for clinical diagnosis of PTSD.

## Data Availability Statement

The raw data supporting the conclusions of this article will be made available by the authors, without undue reservation.

## Ethics Statement

The animal study was reviewed and approved by the Ethics Committee of Jinzhou Medical University and was performed in accordance with the National Guideline on Animal Care.

## Author Contributions

SZ designed the study with FH, YS, and LZ. SZ, JC, and FZ performed MRI examination, immunofluorescence, and behavioral testing. HW, SZ, JC, and XZ performed the data analysis. SZ wrote the manuscript. All authors had reviewed this manuscript critically and approved its final submission.

## Funding

This research was supported by doctoral research startup fund from Department of Science and Technology of Liaoning Province (No. 2019-BS-099), and science and technology fund from Department of Education of Liaoning Province (No. JYTQN2020013).

## Conflict of Interest

The authors declare that the research was conducted in the absence of any commercial or financial relationships that could be construed as a potential conflict of interest.

## Publisher's Note

All claims expressed in this article are solely those of the authors and do not necessarily represent those of their affiliated organizations, or those of the publisher, the editors and the reviewers. Any product that may be evaluated in this article, or claim that may be made by its manufacturer, is not guaranteed or endorsed by the publisher.
